# Incidence of rotavirus infection in children with gastroenteritis attending Jos university teaching hospital, Nigeria

**DOI:** 10.1186/1743-422X-8-233

**Published:** 2011-05-16

**Authors:** Surajudeen A Junaid, Chijioke Umeh, Atanda O Olabode, Jim M Banda

**Affiliations:** 1 Department of Virology, Federal College of Veterinary and Medical Laboratory Technology, National Veterinary Research Institute, Vom, Nigeria

## Abstract

This study was conducted to determine the incidence of rotavirus infection in children with gastroenteritis attending Jos university teaching hospital, Plateau State. A total of 160 children with acute diarrhea were selected by random sampling. Stool samples were obtained and assayed for rotavirus antigens by enzyme linked immunosorbent assay technique using standard diagnostic BIOLINE Rotavirus kit. Demographic data of parents were also recorded. Rotavirus were detected in faeces of 22(13.8%) children with acute diarrhea, 90.9% of positive cases of rotavirus gastroenteritis were under 2 years of age with highest prevalence in children 7-12 months of age. Males excreted rotavirus at a significant higher rate than females (P < 0.05). Rotavirus excretion was highest when all three symptoms (diarrhea, fever and vomiting) occurred in the same child (7.5%) and lower when 2 symptoms occurred together (diarrhea and vomiting) with 3.8%, diarrhea and fever with 1.3% and lowest when diarrhea occurred alone with 1.3%. Playing with toys, attending day care, distance of source of water from toilet, eating of food not requiring cooking and playing with other children may serve as predisposing factors of rotavirus disease in these children.

## Introduction

Rotavirus is recognized as a major cause of non bacterial gastroenteritis (infection of the stomach and intestinal tract leading to diarrhea and vomiting) especially in infants and young children worldwide [1,2]. Almost all children have had a rotavirus infection by the time they are 5 years old [3]. Rotavirus has also been implicated as an etiological agent of diarrhea in older children, adult human, young and adult animals, including calves and piglets. Among rotaviruses are the agents of human infantile diarrhea, Nebraska calf diarrhea, and epizootic diarrhea in infant mice [4].

Rotavirus infection is also known as infantile diarrhea (since almost all children are infected in there first few years of life when they are especially at risk for the disease) or winter diarrhea (because in the United states, the disease occurs more often during winter and springs with the most activity occurring from November to May). It is a highly contagious and unpredictable disease and may lead to severe dehydration (loss of body fluid) and even death [5]. Rotavirus is endemic worldwide; the infection is associated with high rates of morbidity throughout the world and high rates of mortality in developing countries. In the United states, rotavirus infection are responsible for approximately 3 million cases of diarrhea and an estimated 55,000 hospitalization for diarrhea and dehydration in children under 5 years old each year, although these infection cause relatively few deaths in united States. However, in developing countries, rotavirus gastroenteritis account for more than 800,000 childhood deaths per year due to poor nutrition and health care [6].

Rotavirus was coined from the Latin word (rota - meaning wheel), and is given because the viruses have a distinct wheel like shape. The genome of rotavirus consists of 11 segments of double stranded linear molecules of RNA which are 18,555 nucleoside base pair in total. The RNA is surrounded by a double icosahedral protein capsid, the viral particles measures 60-80 nm in diameter and is not enveloped [7].

Rotavirus gastroenteritis is a mild to severe disease, once a child is exposed to rotavirus infection, it takes about 1-2 days (incubation period) before symptoms often starts with fever, nausea and vomiting, which are often followed by abdominal cramps and frequent watery diarrhea (which may last for 3-8 days) infected children may also have a cough and runny nose. Diarrhea especially when it occurs along with vomiting can quickly lead to dehydration in babies and young children. Signs of dehydration include thirst, irritability, restlessness, lethargy, sunken eye, dry mouth and tongue, dry skins, fewer trips to the bath room to urinate, and in infants fewer than 3 wet diapers in 24 hours, lack of interest in playing and extreme sleepiness, child may be sleepy that he or she may be difficult to wake up, fast breathing and rapid heart beat, and a sunken soft spot (fontanel) on top of the head. Dehydration is a serious complication of rotavirus, if not immediately managed it can lead to hypovolemia (a disorder in which the volume of circulating blood decreases) and circulatory collapse and eventual death, in severe cases children may suffer from symptoms of rotavirus gastroenteritis for up to 9 days and then recover [8]. A child may have rotavirus gastroenteritis more than once because there are many different rotavirus types but repeat infection tends to be less severe than the original infection. Asymptomatic infections occur by either subsequent infection of individuals whose immune system confers protection with rotavirus infection with Seroconversion [8].

Rotavirus are stable in the environment and have been in estuary samples, they are so infectious that they can survive for long period of time on toys and ordinary surfaces found in most homes and rotavirus is relatively resistant to most soaps and disinfectants thus preventing your child from exposure can be difficult [7].

Rotavirus is transmitted from person to person through the fecal-oral route. This occurs when the viruses found in stool of infected child is swallowed by another child, in other words children become infected if they put their finger in there mouth after touching something such as toys, books, clothing e.t.c., that has been contaminated by stool of an infected person, this usually happen when children forget to wash their hands after using the toilet or before eating [3]. Health care and child care workers also spread the disease if they don't wash their hands after changing diapers, frequent hand washing is the best tool to limit the spread of rotavirus infection. Rotavirus may also be transmitted through in take of faecally-contaminated water or food which usually occur when infected food handlers who prepare, salad, sandwiches, carrots and other foods that requires no cooking can spread the disease. It may also be transmitted by respiratory droplets that people sneeze, cough, drip, or exhale [7]. Nosocomial transmission is frequent in pediatric wards and hospitals with poor sewage water treatment and sanitation [4].

Diagnosis of rotavirus infection is based on the identification of rotavirus in faeces or suspension of rectal swab collected early in the illness. The virus in stool may be directly demonstrated by electron microscopy, polyacrylamide gel electrophoresis with silver stain. Human rotavirus can also be isolated from stool samples in primary monkey kidney cells. Rapid serological test involve the use of latex agglutination kits while confirmatory test with Enzyme Linked Immunosorbent Assay (ELISA) test for rotavirus specific antigens is also used [[4] and [9]].

The onset of maximum susceptibility correlates with the decline of maternally acquired factors which typically disappears around 5 months, thus susceptibility to disease continues through out life with the most severe disease associated with the infants' first infection. Boys have been found to be twice susceptible and likely to be admitted to hospitals than girls [8]. Adult infections have been reported in military population, hospital workers, travelers and most commonly in parents of infected infants, infecting approximately 50% of parents, one third of these adult infection are asymptomatic, though reinfection occurs in both children and adult [3].

### Justification

Rotavirus infection is not routinely diagnosed in most Nigerian hospitals probably due to the cost of its diagnosis and its clinical spectrum of signs and symptoms are similar to other gastroenteritis, also certain environmental, climatic, sanitary and behavioral factors are associated with the infection. Hospitals may serve as a source for the spread of rotavirus infection leading to nosocomial transmission of the virus. Here lies the justification for this work.

The overall findings of this study suggest that rotavirus is one of the major etiological agents of diarrhea seen in infants and younger children in Jos, Nigeria.

## Materials and methods

### Study area/population

The research was carried out in Jos city, state capital of Plateau State. The research work was conducted at Jos University Teaching Hospital, Plateau State, in two wards, (1) Pediatric ward (2) General out patient department. Children aged 0-5 years with gastroenteritis attending the hospital were chosen as the study population.

### Exclusion criteria

Children with drug history of immuno-suppressive or documented significant back-ground disease such as immuno-deficient syndromes were excluded from the study population.

### Ethical consideration

The ethical clearance for this research was granted by the Jos University Teaching Hospital (JUTH) ethical committee after due processes had been followed. Before the collection of samples, information regarding the study was explained to the parents of the participating children. Oral and written consent for participation in the study was obtained.

### Sample collection

A total of 160 stool samples were collected from children who fall within the age range of 0-5 years with gastroenteritis, after each patient was examined by a physician, the stool samples were collected using a sterile wide mouth universal containers and these container were covered and labeled accordingly. Questionnaires were also administered and filled by the parents who accompanied these children to the hospital. Between 4-5 mls of diarrheic stool samples were collected from each child.

### Transportation of samples

The labeled stool samples were transported on ice to the Virology department of the FCVMLT (National Veterinary Research Institute) N.V.R.I., Vom where they were stored at -20°C until assayed, for the detection of rotavirus antigen by commercially available SD (standard diagnostics) Bioline Rotavirus kit.

### The test

The test was performed as recommended by the manufacturer.

The test was carried out using ELISA method. ELISA is a sensitive and reliable procedure in the detection of antigens to rotavirus. The ELISA kit used for this test was prepared and manufactured by STANDARD DIAGNOSTICS, INC.156-68 Hagal-dong, Korea.

Tel: 82-31-899-9700. http://www.standardia.com.

### Principle of the test

The test utilizes two kinds of antibody in a solid phase sandwich immunochromatography to detect group specific antigens, which include the major inner capsid protein present in rotaviruses.

Nitrocellulose-based membrane pre-coated with rabbit monoclonal anti-rotavirus antibodies and the specially-selected mouse monoclonal anti-rotavirus antibodies are used as detector materials, respectively. Present in human faecal specimens, and then this mixture will react specifically with the rabbit anti-rotavirus antibody in the membrane [[10] and [11]].

### Procedure for the test

The following were procedures observed during the test.

#### 1) Specimen collection

- Test devices and extracted samples were allowed to attain room temperature prior to testing.

- A disposable dropper was used to add 1 ml of sample diluent into a sample collection tube.

- A portion of the feces (about 50 mg) were collected from the stool sample that presented the most secretion under visual inspection using a sterile sample collection swab.

- The swab was inserted into a sample collection tube which contained 1 ml of sample diluent and swab was swirled for at least 10 times to make a mixture.

#### 2) Specimen Transportation and storage

- No transport media were used to store and transport specimens as this may interfere with the test. Faecal specimens were refrigerated at 2-8°C for 72 hours.

#### 3) Test proper

- Test devices and extracted samples were allowed to attain room temperature prior to testing.

- The test device was placed on a flat, dry surface.

- About 3-4 drops of the mixture were added into the sample well marked (S) of test device.

- It was incubated for 10-20 minutes at room temperature and result was read.

### Interpretation of the test

A colour band appears in the left section of the result window to show that the test is working properly (This band is called the Control band). The right section of the result window indicates the test result, and if a colour band appears in this window (This band is called the Test band).

#### Negative result

Only the Control band was present in the test window.

#### Positive result

The Control band and the Test band were present in the test window.

#### Invalid result

The Control band or both the Control band and the test band were not seen leading to an invalid result. Which may be as a result of not following the directions correctly or expired kit, such specimens were retested using new test kit.

### Internal quality control

The SD BIOLINE rotavirus test device has test line and control line on the surface cassettes which are not visible before applying any samples. The Control line is used as procedural control. Control line always appeared when test procedure was performed properly and when test reagent of Control line working.

### Precision, sensitivity and specificity of test

#### Precision

Within run precision was determined using triplicates of 17 different specimens containing concentrations of rotavirus antigens. The negative and positive values were correctly identified 100% of the time.

#### Sensitivity

The limit of detection; the smallest amount of target marker that can be precisely detected have been equal or superior to a leading commercial rotavirus detection EIA kit.

#### Specificity

No cross reactivity test with bacterial and viral panel on SD BIOLINE Rotavirus test as follows; *Escherichia coli, Enterococcus faecalis*, *Poliovirus, Adenovirus *[11].

### Limitations of the test

- Failure to detect rotavirus may be as a result of factors such as collection of specimens at an improper time in the disease when few virions are present and improper sampling and handling of specimens.

- The test cannot be used to differentiate between serotypes of rotavirus.

- A positive result does not preclude the presence of other enteric pathogens, thus concurrent infection with other microbial pathogens is possible and as such additional microbiological tests should be performed in parallel with SD BIOLINE rotavirus test in order to exclude other possible causes of the illness.

- Test results should be interpreted in conjunction with information available from epidemiological studies, clinical assessment of the patient and other diagnostic procedures.

- Some of the parents may provide wrong information or may not fill the questionnaire at all and this will affect the result analysis.

### Benefits of test to patients

Oral re-hydration therapy (ORT) was provided to participating children and also advice on how to identify signs of dehydration and its control were given out free of charge to parents and guardians.

## Result

### Response rate

A total of 160 questionnaires were distributed to the children screened and all the 160 questionnaires were answered and returned, indicating a 100% response rate.

### Data analysis and presentation of finding:-

The incidence of rotavirus infection among children with gastroenteritis screened is as shown in Table 1. It revealed that 22(13.8%) out of the 160 children tested were positive for rotavirus excretion, whereas, 138(86.2%) tested negative for rotavirus excretion. Rotavirus incidence distribution in relation to sex and their statistical analyses are stated in Table 2. It indicated that a total of 64(40%) male and 96(60%) female cases of gastroenteritis were examined in this study. Rotavirus excretion in male cases was 14(8.8%) and female cases 8(5.0%) had parents who attended tertiary, secondary, primary education and none respectively. 4(2.5%), 12(7.5%), 6(3.8%), 0(0%) represents rotavirus ELISA-positive children whose parents were civil servants, business women/men, house wife and others respectively. The highest incidence of rotavirus infection was seen in children whose parents were business women/men. The educational status of the parents of rotavirus positive children revealed that the highest incidence was observed among children whose parents had secondary school level or no formal education. Children whose parents were married had the highest incidence of 18(11.3%), compared to single parents and divorced parents with 2(1.3%) each. Table 3 indicated that children within the age bracket of 7-12 months had the highest rate of infection with 12 (7.5%), while those within the age bracket of 0-6 and 25-60 months had the least with 2(1.3%). Children whose parents were involved in monogamy type of marriage had the highest incidence of 10(6.3%) positivity while those whose parents who were involved in polygamy had 8(5.0%) out of the total number of children enrolled (Table 4). A comparison of clinical variables between children with or without rotavirus infection is shown in Table 5. Playing with toys, attendance of day care/nursery school and eating of foods that do not require cooking appeared to serve as the major risk factors for rotavirus infection, with P values 0.011, 0.013 and 0.039 respectively, as shown in Table 6.

**Table 1:** Frequency of rotavirus infection among children with gastroenteritis screened.

Rotavirus status	Frequency	Percentage (%)
No of positive samples	22	13.8
No of negative samples	138	86.2

Total	160	100.0

**Table 2:** Rotavirus incidence distribution in relation to sex

Sex	Rotavirus status of children			
			
	Positive	Negative	Total	P-value
Male count	14	50	64	
% of total	8.8%	31.3%	40.0%	
Female count	8	88	96	0.015
% of total	5.0%	55.0%	60.0%	

Total	22	138	160	
% of total	13.8%	86.3%	100.0%	

**Table 3:** Rotavirus incidence distribution in relation to age grouping

Age group of children (months)		Rotavirus status of Children			
				
		Positive	Negative	Total	P-value
0-6	count	2	8	10	
	% of total	1.3%	5.0%	6.3%	
7-12	count	12	40	52	
	% of total	7.5%	25.0%	32.5%	0.047
13-24	count	6	54	60	
	% of total	3.8%	33.8%	37.5%	
25-60	count	2	36	38	
	% of total	1.3%	22.5%	23.8%	

Total	count	22	138	160	
	% of total	13.8%	86.2%	100.0%	

**Table 4:** Rotavirus incidence distribution in relation to demographic characteristics of parents/guardians.

Variables (Parents/guardians)	Rotavirus status of children			
			
	Positive (%)	Negative (%)	Total(%)	P-value
Educational background				

Tertiary education	2(1.3)	1610.0)	18(11.3)	
Secondary education	8(5.0)	38(23.7)	46(28.8)	0.677
Primary education	4(2.5)	18(11.3)	22(13.8)	
None	8(5.0)	66(41.2)	74(46.2)	

Total(%)	22(13.8)	138(86.2)	160(100.0)	

Occupation				

Civil servant	4(2.5)	32(20.0)	36(22.5)	
Business women/men	12(7.5)	70(43.7)	82(51.3)	0.871
House wife	6(3.8)	36(22.5)	42(26.2)	
Others	0(0.0)	0(0.0)	0(0.0)	

Total(%)	22(13.8)	138(86.2)	160(100.0)	

Marital status				

Single	2(1.3)	6(3.8)	8(5.0)	
Married	18(11.3)	120(75.0)	138(86.3)	0.633
Divorced	2(1.3)	12(7.4)	14(8.7)	

Total(%)	22(13.8)	138(86.2)	160(100.0)	

Type of marriage				

Polygamy	8(5.0)	46(28.7)	54(33.8)	0.595
Monogamy	10(6.3)	74(46.3)	84(52.5)	

Total(%)	18(11.3)	120(75.0)	138(86.3)	

**Table 5:** The spectrum of signs and symptoms between children with or without rotavirus infection.

Variables	Positive (%) (n = 11)	Negative (%) (n = 69)
Vomiting only	6(27.3)	32(23.2)
Fever only	2(9.1)	16(11.6)
Fever and vomiting	12(54.5)	56(40.6)
Without fever and vomiting	2(9.1)	34(24.6)
Upper respiratory infection	8(8.0)	130(92.0)
Frequency of diarrhea/day(mean)	4.2	5.5
Duration of diarrhea/days(mean)	5	5.8
Appearance:-		
Watery	16(72.7)	96(69.6)
Mucoid	6(27.3)	38(27.5)
Bloody	0(0.0)	4(2.9)

**Table 6:** Rotavirus incidence distribution in relation to possible risk factors.

Variables	Rotavirus status of children			
			
	Positive(%)	Negative(%)	Total(%)	P-value
Heard of rotavirus				
Infection before				

Yes	0(0.0)	0(0.0)	0(0.0)	0.059
No	22(13.8)	138(86.2)	160(100.0)	

Total(%)	22(13.8)	138(86.2)	160(100.0)	

Source of drinking water				

Tap water	12(7.5)	58(36.7)	70(43.8)	
Well water	8(5.0)	56(35.0)	64(40.0)	0.213
Stream water	0(0.0)	18(11.3)	18(11.3)	
Others	2(1.3)	6(3.8)	8(5.0)	

Total(%)	22(13.8)	138(86.2)	160(100.0)	

Type of toilet used				

Water system	12(7.5)	40(25.0)	52(32.5)	
Pit toilet	8(5.0)	88(55.0)	96(60.0)	
Bucket system	2(1.3)	10(0.0)	12(0.0)	0.443
In bushes	0(0.0)	0(0.0) 0(0.0)		
Others		0(0.0)	0(0.0)	

Total(%)	22(13.8)	138(86.2)	160(100.0)	

Distance of toilet from water source				

Far	16(10.0)	80(50.0)	96(60.0)	0.043
Near	6(3.8)	58(36.3)	64(40.0)	

Total(%)	22(13.8)	138(86.2)	160(100.0)	

Playing with toys				

Yes	20(12.5)	122(76.3)	142(90.0)	0.011
No	2(1.3)	16(10.0)	18(11.3)	

Total(%)	22(13.8)	138(86.2)	160(100.0)	

Wash child's hands after every visit to toilet				

Yes	10(6.3)	28(23.8)	48(30.0)	0.089
No	12(7.5)	100(63.8)	112(71.2)	

Total(%)	22(13.8)	138(86.2)	160(100.0)	

Wash child's hands before every meal				

Yes	16(10.0)	110(68.8)	126(78.8)	0.457
No	6(3.8)	28(17.5)	34(21.3)	

Total(%)	22(13.8)	138(86.2)	160(100.0)	

Attends day care or nursery school				

Yes	16(10.0)	52(32.6)	68(42.6)	0.013
No	6(3.8)	86(53.8)	92(57.5)	

Total(%)	22(13.8)	138(86.2)	160(100.0)	

Consumption of food that need no cooking				

Yes	6(3.8)	66(41.3)	72(45.0)	0.039
No	16(7.5)	72(45.0)	88(55.0)	

Total(%)	22(13.8)	138(86.2)	160(100.0)	

Often play with other people/children				

Yes	8(5.0)	52(32.5)	60(37.5)	0.041
No	14(8.8)	86(53.8)	100(62.5)	

Total(%)	22(13.8)	138(86.2)	160(100.0)	

No. of times house is cleaned per week				

Once	0(0.0)	0(0.0)	0(0.0)	
Twice	2(1.3)	10(6.3)	12(7.5)	0.760
Trice or more	20(12.5)	128(80.0)	148(92.7)	
None	0(0.0)	0(0.0)	0(0.0)	

Total(%)	22(13.8)	138(86.2)	160(100.0)	

## Discussion

Rotavirus most often infects infants and young children and it is one of the most common causes of viral gastroenteritis [3].

Our findings revealed that rotaviruses are important aetiologic agents of acute diarrhea, accounting for over one eighth of all cases with acute gastroenteritis, with an incidence of 13.8%. The incidence of 13.8% observed in our study is comparable with reports from Ile-Ife, Nigeria (13.8%) and Jamaica (15.0%) [[12] and [13]].

Most of the infected children in our study were under 2 years of age, with highest prevalence between 7-12 months (X^2 ^= 36.01, P < 0.05). This age distribution is comparable to previous reports in Iran [14], and Ile-Ife, Nigeria [15]. This is in accordance with the assumption that in under-developed areas the early peak of rotavirus diarrhoea may result from early exposure to contaminated sources as well as over-crowded homes, more so, since almost all humans experience at least one rotavirus infection by 3 years of age and circulating rotavirus antibodies remain detectable indefinitely [16]. This may lead to protection against rotavirus infection and disease or at least milder forms of disease, which result in lower rate of rotavirus gastroenteritis in older children. The low incidence rate of 1.3% in children within 0-6 months which is similar to report from Guinea-Bissau [17] may be due to passive immunity acquired by the infants from their mothers which wades off after 6 months since it is passively acquired, and also possible, is the higher rate of breast feeding in the age group which also protects the infants via passing of IgA anti-rotavirus antibodies to the infants [14].

Detection of rotavirus with higher numbers in males than in females was noted in our study, this indicated that males excreted significant high rate of rotavirus in their faeces than their female counterparts (X^2 ^= 2.97, P < 0.05). The ratio of the infection of male to female children was (1.8: 1) and is in contrast to the reported ratios of 1.5: 1 by Samir *et al *from Bahrain and 1: 2.4 by Puri *et al *from India [18]. This is in agreement with the finding of [8] that boys have been found to be twice susceptible and likely to be admitted to hospitals than girls. Whether this difference is due to sex susceptibility or by chance is however questionable and needs further investigation, while the reason(s) for the difference in ratio occurrence within different geographical location is not well understood.

Vomiting followed by fever appears to be more common with rotavirus diarrhea, the significant difference between rotavirus ELISA positive children and rotavirus ELISA negative children was the presence of all 3 symptoms: - Diarrhoea, fever and vomiting (X^2 ^= 5.41, P < 0.05), the same was observed in the study conducted by [12]. The development of upper tract symptoms such as coryza (runny nose) and cough were not statistically significant (P > 0.05), which is similar to [3] who reported that upper respiratory infection may occur in infected children.

The demographic data (educational background, occupation, marital status, type of marriage) of the parents/guardians of these children assayed were also linked with the status of rotavirus infection within the children. Children whose parents were married and business men and women had the highest prevalence of rotavirus excretion, while parents who had attended secondary education and those not educated had children with the highest incidence of rotavirus infection (5.0%) each. These observation was not statistically significant (P > 0.05), indicating that the observed differences may be due to chance and not a certainty and this implies that rotavirus infects children regardless of parent's demographic characteristics.

The possible risk factors associated with rotavirus infection was found to be associated with children using toys (P < 0.05). These toys can be easily contaminated by older children who may be asymptomatic carriers of the virus in their finger nails, hands etc, children are seen to put objects into their mouths while playing or scratching their gums when they are about to start growing teeth, such contaminated objects then serve as source of infection to them [3]. The relative frequency of infected children was higher in children attending school (day care centre) in comparison with other subjects (P < 0.05), this may be attributed to higher risk of virus transmission in day care centers due to more close contact in these environments or probably lower rate of breast feeding in these children [18]. Other risk factors such as distance of toilet from water sources, consumption of food that do not require cooking e.g salad, sandwiches and even carrots, playing with other children often, were all observed to be associated with the incidence of rotavirus infection (P < 0.05).

## Conclusion

The overall findings of this study showed that rotavirus is one of the major etiological agents of diarrhea seen in infants and younger children. Rotavirus infection was prevalent in 7-12 months old children with males more susceptible to rotavirus infection than females. Rotavirus detection was greatest when diarrhea, vomiting and fever occurred together and lowest when each symptoms occurred alone, and possible risk factors to rotavirus infection include playing with other children, distance of water sources from toilet, attending of day care centers, and playing with toys or consumption of food that do not require cooking.

The strategies for rotavirus control include identifying the target population for rotavirus vaccination, educating parents on how to identify and recognize the signs of dehydration and also to know that rotavirus infection in children is unavoidable and should be looked out for [17]. However, the significant higher prevalence in children attending day care emphasizes the need to pay attention to the role of child care as an important factor in the epidemiology of rotavirus gastroenteritis.

## Recommendations

1) Further investigations are needed to provide a more accurate picture of epidemiology of rotavirus disease and also its serotypes in Jos. This is highly needed to design an effective vaccination against rotavirus in future.

2) Improvement of rotavirus vaccines and development of alternative vaccines should continue and then administered to children in developing countries to lower the incidence rate.

3) Laboratory workers should assay for rotavirus in diarrhea cases when bacterial and parasitic agent assays all show negative results.

4) To understand the reasons for the observed different findings of rotavirus infection and the seasonal pattern, conducting studies covering a longer period of time is essential.

## Competing interests

The authors declare that they have no competing interests.

## Authors' contributions

JSA and UC participated in the design of the study and performed the investigation, analysis and interpretation of data. OAO and BJM participated in the design and coordination. JSA and UC drafted the manuscript while JSA also working as the corresponding author. All authors read and approved the final manuscript.
